# Comment on “Intravenous lidocaine infusion accelerates postoperative bowel function recovery in patients undergoing lumbar surgery: a multi-center, randomized controlled trial”

**DOI:** 10.1097/JS9.0000000000004541

**Published:** 2025-12-09

**Authors:** Xuesong Yin, Randong Peng, Yanjun Zhang

**Affiliations:** a College of Clinical Traditional Chinese Medicine, Gansu University of Chinese Medicine, Lanzhou, Gansu, China; b Orthopaedics Department, Gansu Provincial Hospital of Traditional Chinese Medicine, Lanzhou, Gansu, China

*Dear Editor,*

We read with considerable interest the article entitled “Intravenous Lidocaine Infusion Accelerates Postoperative Bowel Function Recovery in Patients Undergoing Lumbar Surgery: A Multi-center, Randomized Controlled Trial” published in your prestigious journal[1]. Lumbar surgery is often associated with significant postoperative pain, high opioid consumption, and delayed bowel function recovery. The authors have thoughtfully explored lidocaine as an adjunct to multimodal analgesia to accelerate postoperative bowel function recovery and reduce intraoperative opioid usage. We commend the researchers for conducting a well-designed multicenter randomized controlled trial that contributes valuable data to this field. However, several key considerations warrant discussion to strengthen the interpretation of the findings and inform future clinical practice.

First, the study inferred that lidocaine concentrations were maintained within the therapeutic window of 1–3 µg/ml, relying exclusively on pharmacokinetic data from the original LOLIPOP trial[2]. However, the original trial population consisted of children aged 2–12 years, representing a group vastly different from the elderly cohort in this study. The elderly population generally exhibits reduced metabolic capacity and age-related physiological declines in hepatic and renal function, meaning pharmacokinetic profiles are not entirely consistent with those observed in pediatric populations. Furthermore, plasma concentration serves as a critical marker for determining whether lidocaine achieves an effective dose and whether toxicity risks exist[3]. The absence of direct plasma concentration monitoring in this study undermines the causal link between lidocaine administration and accelerated postoperative bowel function recovery. Therefore, we recommend incorporating lidocaine plasma concentration monitoring into the design of future trials.

Second, the study failed to explicitly document surgical details and procedural complexity across the two groups, such as differentiation between single- vs. multi-level procedures, or between lumbar discectomy and fusion surgeries. Additionally, details regarding antibiotic use or other medications with potential effects on intestinal function were not reported. Such factors may independently impact postoperative bowel recovery and opioid consumption[4], and their omission could introduce residual confounding stemming from baseline imbalances between groups. Addressing these gaps in future trials would strengthen the clinical applicability of the results.

Third, while the authors reported adverse events such as dizziness, postoperative nausea and vomiting, sinus bradycardia, and hypotension, they did not assess other common lidocaine-associated adverse effects, including central nervous system toxicity (e.g., somnolence, seizures, tinnitus) or local venous irritation[5]. Furthermore, the severity of adverse events could have been graded using the Clavien–Dindo classification system to comprehensively evaluate lidocaine’s safety profile in elderly lumbar surgery patients[6].

Finally, the article states: “In cases of breakthrough pain (Numerical Rating Scale, NRS ≥ 4), patients received oral tylophorin (oxycodone 5 mg plus acetaminophen 375 mg), or intramuscular fentanyl (0.05 mg) or morphine (5 mg), followed by additional analgesia per protocol requirements.” However, it is unclear whether this rescue analgesia protocol was actually utilized and, if so, with what frequency. Had there been discrepancies in the rate of rescue analgesic administration between the two groups, this might have indirectly impacted bowel function recovery. Numerous studies have shown that rescue opioid administration can delay gastrointestinal motility[7], yet the study did not report this critical intermediate variable—precluding the ability to rule out its confounding influence on the primary outcome.

In conclusion, this study offers promising evidence that intravenous lidocaine administration may accelerate postoperative bowel function recovery and reduce intraoperative opioid requirements in elderly patients undergoing lumbar spine surgery. We greatly appreciate the authors’ contributions and anticipate larger-scale trials to yield more robust evidence in this area.

We declare that the above complies with the TITAN guideline, which mandates openness in artificial intelligence reporting[8].

## Data Availability

Data availability is not applicable to this article, as no new data were created or analyzed in this study.

## Declarations

Artificial intelligence (AI) tools were used only for language polishing (grammar and style). Specifically, ChatGPT 5.0 was employed on 25 November 2025, with default settings. No AI tools were used for data analysis, interpretation, or generation of scientific content. The authors confirm that they take full responsibility for the integrity and accuracy of the final manuscript.
